# Bone Strength of the Calcaneus Is Associated with Dietary Calcium Intake in Older Japanese Men, but Not Women

**DOI:** 10.3390/nu14245225

**Published:** 2022-12-08

**Authors:** Keita Suzuki, Hiromasa Tsujiguchi, Akinori Hara, Sakae Miyagi, Thao Thi Thu Nguyen, Yasuhiro Kambayashi, Yukari Shimizu, Fumihiko Suzuki, Chie Takazawa, Masaharu Nakamura, Hirohito Tsuboi, Takayuki Kannon, Atsushi Tajima, Hiroyuki Nakamura

**Affiliations:** 1 Department of Hygiene and Public Health, Faculty of Medicine, Institute of Medical, Pharmaceutical and Health Sciences, Kanazawa University, Kanazawa 920-8640, Ishikawa, Japan; 2 Department of Public Health, Graduate School of Advanced Preventive Medical Sciences, Kanazawa University, Kanazawa 920-8640, Ishikawa, Japan; 3 Kanazawa University Advanced Preventive Medical Sciences Research Center, Kanazawa 920-8640, Ishikawa, Japan; 4 Innovative Clinical Research Center, Kanazawa University, Kanazawa 920-8640, Ishikawa, Japan; 5 Department of Epidemiology, Faculty of Public Health, Haiphong University of Medicine and Pharmacy, Hai Phong 04000-05000, Vietnam; 6 Department of Public Health, Faculty of Veterinary Medicine, Okayama University of Science, Imabari 794-0085, Ehime, Japan; 7 Department of Nursing, Faculty of Health Sciences, Komatsu University, Komatsu 923-8511, Ishikawa, Japan; 8 Community Medicine Support Dentistry, Ohu University Hospital, Koriyama 963-8611, Fukushima, Japan; 9 Graduate School of Human Nursing, The University of Shiga Prefecture, Hikone 522-8533, Shiga, Japan; 10 Department of Biomedical Data Science, School of Medicine, Fujita Health University, Toyoake 470-1101, Aichi, Japan; 11 Department of Bioinformatics and Genomics, Graduate School of Advanced Preventive Medical Sciences, Kanazawa University, Kanazawa 920-8640, Ishikawa, Japan

**Keywords:** calcium intake, bone strength, grip strength, older people, cross-sectional study

## Abstract

The relationship between calcium intake and bone strength in older Asian individuals, including Japanese, is controversial; therefore, we herein investigated this relationship in older Japanese populations. We performed a cross-sectional analysis of 314 participants older than 65 years who voluntarily participated in a medical examination and responded to questionnaires. The osteo-sono assessment index (OSI) measured at the right calcaneus using a quantitative ultrasonic device was used as an indicator of bone strength. The daily dietary intake of calcium was assessed using a brief-type self-administered diet history questionnaire. A two-way analysis of covariance revealed a significant interaction between sex and calcium intake on the OSI (*p* < 0.01). A multiple regression analysis showed a positive correlation between calcium intake and the OSI in males (*p* < 0.01), but not females (*p* = 0.27). In females, grip strength divided by body weight positively correlated with the OSI (*p* = 0.04). The present results suggest that a higher calcium intake contributes to bone strength in older Japanese males. Although a higher grip strength may contribute to bone strength in females, the potential of estrogen as a confounding factor needs to be considered.

## 1. Introduction

Health care providers and the general population consider a sufficient calcium intake to be an essential factor for maintaining or improving bone strength. The Food and Nutrition Board at the National Academies of Sciences, Engineering, and Medicine recommends a calcium intake of 1200 mg/day to promote bone maintenance and a neutral calcium balance in individuals aged 70 years and older [1].

However, not all studies support the beneficial effects of an adequate dietary calcium intake on bone. A systematic review that included longitudinal cohorts concluded that a positive relationship was not demonstrated between calcium intake and changes in bone mineral density (BMD) in women aged 60 years and older [2]. Furthermore, a meta-analysis of randomized controlled trials revealed that an increased calcium intake from dietary sources or by taking calcium supplements achieved only small non-progressive increases in BMD in participants aged 50 years or older [3]. Moreover, a longitudinal cohort composed of community-based, multiethnic women across the menopause transition recently reported that the consumption of dairy products did not contribute to the preservation of BMD or prevention of fractures [4]. Therefore, the benefits of calcium intake for maintaining BMD appear to be unestablished.

Serum 25-hydroxyvitamin D (25(OH)D) concentrations influence the absorption efficiency of calcium in the intestines [5,6]. Previous studies demonstrated that calcium absorption varied with age [7,8] and race [9]. Therefore, the effects of calcium consumption on BMD may differ according to race, age, and the vitamin D status. However, the majority of studies in reviews [2,3] recruited Caucasians and Blacks. Moreover, the age range of participants in each study was broad and did not focus on the elderly. To the best of our knowledge, only a few studies have investigated the relationships between dietary calcium intake and bone strength parameters in an older community-based Asian population [10,11,12], and the relationships observed were neither significant nor consistently linear.

Therefore, we herein examined a general Japanese population aged 65 years and older in which calcium absorption is considered to be reduced due to aging. Since our cohort resides in a rural area in Japan, high ethnic homogeneity was expected due to the lack of immigrants from other countries in the region. The aim of this cross-sectional analysis was to investigate the relationship between dietary calcium intake and bone strength in an older Japanese population according to sex.

## 2. Methods

### 2.1. Study Design and Participants

We utilized cross-sectional data collected between 2011 and 2015 in the Shika study. We have conducted a longitudinal community-based observational study since 2011 among the residents of Shika town, which is located on the Noto peninsula in Ishikawa prefecture, Japan. The population of Shika town is almost 20,000, and more than 40% are older than 65 years. In the Shika study, self-administrated questionnaires were distributed to all adults older than 40 years in the four model districts by trained interviewers. The interviewers were instructed on the outline of the Shika study and how to fill in the questionnaires before their distribution. Moreover, an invitation letter for comprehensive medical examinations was distributed to the residents in the four districts. Participants voluntarily underwent a medical examination.

In the present study, we included participants older than 65 years who responded to the questionnaires and underwent the medical examination (*n* = 620). Participants with missing data on variables used in the analysis were excluded. To avoid a treatment bias, participants who received treatments for osteoporosis were omitted. Data collected from 314 participants who completed the medical examination and replied to the questionnaires were available for analysis in the present study.

The present study was approved by the Medical Ethics Committee of Kanazawa University (approval number 1491). All participants provided written informed consent for inclusion before participation. The present study was conducted in accordance with the Declaration of Helsinki.

### 2.2. Assessment of Nutrient Intake

A brief-type self-administered diet history questionnaire (BDHQ) was used to assess the daily dietary intakes of protein, lipids, carbohydrates, vitamin D, calcium, and total energy. Participants were asked about the consumption frequency of 58 food and beverage items during the previous month in BDHQ. These items are mainly from the food list used in the National Health and Nutrition Survey of Japan and are commonly consumed in Japan. The validity of BDHQ and a satisfactory ranking ability for many nutrients in Japanese individuals were previously demonstrated [13,14]. We omitted participants who reported a total energy intake of less than 600 kcal/day (half of the energy intake required for the lowest physical activity category) or more than 4000 kcal/day (1.5-fold the energy intake required for the moderate physical activity category) from the analysis because of under-/overestimations of nutrient intake. Nutrient data were adjusted for daily energy intake using the density method. The participants were divided into two groups according to the median calcium intake calculated in males and females, respectively.

### 2.3. Measurement of Bone Strength

A previous study reported a strong correlation between the osteo-sono assessment index (OSI) measured at the calcaneus and BMD measured by dual-energy X-ray absorptiometry (DXA) at the radius [15]. To calculate the OSI, the speed of sound (SOS) and the transmission index (TI) were measured at the right calcaneus using a quantitative ultrasonic device (AOS-100NW-B, Hitachi Aloka Medical, Tokyo, Japan). The SOS reflects the ultrasound velocity that penetrated the calcaneus. The higher SOS indicates a higher bone density. The TI indicates the frequency-dependent attenuation of the ultrasound. The greater bone mass shows a higher TI [16]. The OSI was given by the following formula [17]:

OSI = TI × SOS^2^ (1)

In the present study, the participants were assigned into two groups according to the median of the OSI calculated for each sex.

### 2.4. Assessment of Other Variables

Age, height, weight, blood pressure, grip strength, and calf circumference were assessed in the medical examination. We calculated body mass index (BMI) as weight in kilograms divided by the square of height in meters. Calf circumference and grip strength were divided by body weight (GS/BW) to adjust for the influence of body size. Fasting plasma glucose (FPG), serum cystatin C, serum calcium, serum 25(OH)D concentrations, and intact parathyroid hormone (PTH) were measured using blood samples taken in the medical examination. Blood samples were drawn from the forearm vein in the morning after fasting for 12 h. Serum 25(OH)D concentrations were measured using a radioimmunoassay (25-hydroxyvitamin D 125I RIA Kit, DiaSorin Inc., Stillwater, MN, USA). Intact PTH was measured by an electro-chemiluminescence immunoassay (SRL, Inc., Tokyo, Japan). Estimated glomerular filtration rates (eGFR) based on cystatin C were calculated using the following equations modified for the Japanese population [18]:

eGFR for males: [104 × (serum cystatin C)^−1.019^ × 0.996^age^] − 8

eGFR for females: [104 × (serum cystatin C)^−1.019^ × 0.996^age^ × 0.929] − 8

Drinking habits and the smoking status were assessed using a self-administered questionnaire. To assess drinking habits, participants were asked “How much alcohol do you drink per day?”. Based on their answer, participants were divided into the following four groups: non-drinker, less than 20 g/day, 20 to 40 g/day, and more than 40 g/day. Participants were assigned to non-smoker, ex-smoker, and current smoker groups according to their answer on their smoking status.

### 2.5. Statistical Analysis

Continuous variables were summarized as means and standard deviations (SD), and categorical variables as numbers (*n*) and percentages (%). The mean levels of continuous variables and categorical variables were compared between the low- and high-OSI groups using the Student’s *t*-test or chi-square test. A two-way analysis of covariance (ANCOVA) was used to examine the interaction between sex and calcium intake levels on the OSI with adjustments for age, BMI, GS/BW, eGFR, drinking habits, the smoking status, intact PTH, and the serum 25(OH)D concentrations. The relationship between the serum calcium concentrations and intact PTH was assessed using a multiple regression analysis with adjustments for age, BMI, drinking habits, and the smoking status. A multiple regression analysis was performed to examine the relationship between calcium intake and the OSI according to sex with adjustments for the following independent factors: age, BMI, GS/BW, eGFR, drinking habits, the smoking status, intact PTH, and the serum 25(OH)D concentrations.

All statistical analyses were performed using the Japanese version of IBM SPSS Statistics version 27.0 (IBM Japan, Tokyo, Japan). A two-sided *p*-value < 0.05 was considered to be significant.

## 3. Results

### 3.1. Participant Characteristics

Table 1 shows participant characteristics. Among 314 participants, 157 were males and 157 were females. The mean ages of male and female participants were 71.7 ± 5.8 and 72.1 ± 6.2 years, respectively. The OSI was significantly higher in males than in females (*p* < 0.01), whereas calcium intake was significantly higher in females (*p* < 0.01). Males had significantly higher 25(OH)D concentrations than females (*p* < 0.01), while intact PTH was lower in males than in females (*p* = 0.01).

### 3.2. Comparison of Characteristics between Low- and High-OSI Groups according to Sex

The results of comparisons between the low- and high-OSI groups are shown in Table 2. In males, the high-OSI group was significantly younger than the low-OSI group (*p* < 0.01). The high-OSI group had a significantly higher body weight (*p* < 0.01) and BMI (*p* < 0.01) than those in the low-OSI group, whereas CC/BW was significantly lower (*p* < 0.01).

In females, age (*p* = 0.02) and CC/BW (*p* < 0.01) were significantly lower in the high-OSI group than in the low-OSI group. The high-OSI group showed a significantly higher body weight (*p* < 0.01) and BMI (*p* = 0.02) than those in the low-OSI group.

### 3.3. Interaction between Sex and Calcium Intake on the OSI

Table 3 shows the results of a two-way ANCOVA, which was performed to examine the interaction between sex and calcium intake on the OSI. A significant main effect of sex on the OSI was observed after adjustments for age, BMI, eGFR, GS/BW, drinking habits, the smoking status, intact PTH, and the serum 25(OH)D concentrations (*p* < 0.01), whereas the main effect of calcium intake was not significant (*p* = 0.28). A two-way ANCOVA detected a significant interaction between sex and calcium intake on the OSI after adjustments for the covariates (*p* < 0.01).

### 3.4. Multiple Regression Analysis of the Serum Calcium Concentrations as a Dependent Variable according to Sex

The relationship between the serum calcium concentrations and intact PTH is shown in Table 4. Intact PTH negatively correlated with the serum calcium concentrations in all participants (*p* < 0.01), males (*p* < 0.01), and females (*p* = 0.03). A significant negative association between the serum calcium concentrations and age was observed in all participants (*p* = 0.03).

### 3.5. Multiple Regression Analysis of the OSI as a Dependent Variable according to Sex

The results of a multiple regression analysis are shown in Table 5. Among all participants, the relationship between calcium intake and the OSI was not significant (*p* = 0.14). The OSI positively correlated with BMI (*p* < 0.01), and GS/BW (*p* < 0.01), and negatively correlated with age (*p* = 0.01), and intact PTH (*p* = 0.01). Moreover, the OSI correlated with drinking habits (*p* < 0.01) and the smoking status (*p* < 0.01).

In males, the OSI positively correlated with calcium intake (*p* < 0.01), BMI (*p* < 0.01), and the serum 25(OH)D concentrations (*p* = 0.04), and negatively correlated with age (*p* < 0.01).

In females, calcium intake did not correlate with the OSI (*p* = 0.27). However, the OSI positively correlated with BMI (*p* < 0.01), eGFR (*p* = 0.02), and GS/BW (*p* = 0.04), and negatively correlated with age (*p* < 0.01). A significant association between the smoking status and OSI was observed (*p* = 0.03).

## 4. Discussion

The results of this cross-sectional study demonstrated that a higher dietary calcium intake was associated with a higher OSI in males even after adjustments for covariates. In females, the OSI did not correlate with calcium intake, but positively correlated with GS/BW and eGFR.

PTH regulates calcium homeostasis in the body [19]. The secretion of PTH from the parathyroid glands is altered to keep serum calcium levels within the appropriate range depending on extracellular calcium concentrations. In the present study, a negative correlation between the serum calcium concentrations and intact PTH was observed. It is considered that the secretion of PTH was upregulated in individuals with lower serum calcium concentrations. In addition, PTH regulates bone remodeling by stimulating osteoblasts and indirectly activating osteoclasts [20]. These studies indicate that PTH strongly influences calcium homeostasis and bone metabolism. Therefore, we adjusted the association between calcium intake and the OSI with intact PTH in the present study.

The relationship between the OSI and calcium intake differed between males and females in the present study. A discrepancy in this relationship between males and females was also reported in a previous study [21]. The sex difference observed in the effects of calcium intake on bone strength may be partially explained by differences in serum 25(OH)D concentrations between males and females. Previous studies reported that serum 25(OH)D concentrations were higher in males than in females [22,23]. In the present study, males had significantly higher 25(OH)D concentrations than females. Vitamin D regulates the intestinal absorption of dietary calcium [5,6]. Therefore, calcium absorption appeared to be more inefficient in females. Furthermore, aging has been shown to decrease calcium absorption due to vitamin D insufficiency and intestinal resistance to the effects of vitamin D [7,24]. Therefore, the lack of a relationship between calcium intake and the OSI was attributed to impaired calcium absorption in older females in the present study.

Several previous studies reported that the SOS and broadband ultrasound attenuation (BUA) measured at the calcaneus were closely associated with BMD assessed using DXA [25,26]. In addition, the SOS and BUA negatively correlated with age [19,26]. Therefore, we believe that the SOS and BUA measured by ultrasound reflect the age-related loss of bone density. However, the possibility that the OSI does not rigorously detect the loss of bone density should also be considered. The OSI depends not only on the bone density in the calcaneus but also on the amount of yellow marrow or fat [27]. Furthermore, the impact of marrow on the quantitative ultrasound transmission of cancellous bone is likely to be greatest in women with low BMD [27]. A previous study reported that the amount of marrow fat in the calcaneus does not vary with the development of osteoporosis [28]. Therefore, the lack of significant association of calcium intake with the OSI in females in the present study may be attributed to the inability of the OSI to detect the reduction of bone density particularly in females.

GS/BW correlated with the OSI in females in the present study. Previous studies reported that grip strength positively correlated with BMD in aged adults and postmenopausal women [29,30,31]. Mechanical loading generated by muscle is an essential mechanism for maintaining bone health [32,33]. Therefore, the present results appear to support previous findings and suggest that the maintenance of muscle strength contributes to bone strength in older Japanese females.

However, the relationship between GS/BW and the OSI in females needs to be interpreted with caution because it was not observed in males. Furthermore, the effects of estrogen on bone mass and muscle function need to be considered. An estrogen deficiency in women after menopause activates osteoclastic bone resorption, causing osteoporosis [34]. Furthermore, estrogen treatments ameliorate osteoporosis [35,36]. In addition, a decline in estrogen levels in menopausal women was previously reported to impair muscle strength via the inadequate preservation of skeletal muscle mass and decrements in the quality of the remaining skeletal muscle [37,38]. Therefore, estrogen levels may have mediated the relationship between GS/BW and the OSI in the present study. However, the effects of estrogen remain unclear because we did not measure estrogen levels. Therefore, the potential of estrogen as a confounding factor needs to be considered when interpreting the present results.

There are further limitations that need to be addressed. The causality of the relationship of the OSI with calcium intake and GS/BW remains unclear because of the nature of a cross-sectional study. Moreover, selection bias may limit the generalizability of the present results. The ratio of health-conscious individuals was likely to be high because only voluntary participants underwent the medical examination in the present study. The lack of assessment of the bone density at the femoral neck or lumbar may prevent our findings from providing robust suggestions on the prevention of fractures. However, the previous studies reported that the SOS and BUA measured at the calcaneus closely correlated with BMD of the femoral neck, spine, and total body measured by DXA [39,40,41]. Therefore, although further studies focused on the femoral neck and spine are needed, our findings observed at the calcaneus are likely to apply to the other sites of the body.

## 5. Conclusions

Dietary calcium intake was associated with the OSI in older Japanese males, but not females. On the other hand, GS/BW correlated with the OSI in females. The present results suggest that the effects of calcium intake on the preservation of bone strength may differ between the sexes in an older Japanese population.

## Figures and Tables

**Table 1:** Participant characteristics.

	Total (*n* = 314)		Male (*n* = 157)		Female (*n* = 157)		
	Mean (*n*)	SD (%)	Mean (*n*)	SD (%)	Mean (*n*)	SD (%)	*p*-Value
Age (y)	71.9	6.0	71.7	5.8	72.1	6.2	0.49
Height (cm)	155.2	14.8	162.5	13.1	147.9	12.8	**<0.01**
Weight (kg)	57.6	10.0	63.5	9.2	51.8	6.7	**<0.01**
BMI (kg/m^2^)	23.4	2.9	23.7	3.1	23.2	2.6	**0.10**
Systolic blood pressure (mmHg)	143	19	143	18	142	19	0.92
Diastolic blood pressure (mmHg)	78	11	78	11	77	11	0.28
Fasting plasma glucose (mg/dL)	99.3	19.3	103.0	22.3	95.6	15.1	**<0.01**
Serum calcium concentrations (mg/dL)	9.31	0.32	9.29	0.31	9.33	0.33	0.20
Serum 25(OH)D concentrations (ng/mL)	25.8	7.8	28.0	7.8	23.6	7.2	**<0.01**
Intact PTH (pg/mL)	47.8	18.2	45.1	17.0	50.5	19.0	**0.01**
CC/BW (cm/kg)	0.58	0.07	0.54	0.05	0.63	0.05	**<0.01**
GS/BW (kgf/kg)	0.51	0.11	0.58	0.10	0.44	0.08	**<0.01**
eGFR (mL/min/1.73 m^2^)	71.6	14.8	71.4	15.2	71.8	14.5	0.80
Osteo-sono assessment index	2.54	0.36	2.75	0.34	2.34	0.23	**<0.01**
Total energy intake (kcal/day)	1899	625	2083	627	1715	567	**<0.01**
Protein intake (g/1000 kcal)	15.6	3.5	14.9	3.2	16.3	3.6	**<0.01**
Fat intake (g/1000 kcal)	24.0	6.1	22.4	5.8	25.6	5.9	**<0.01**
Carbohydrate intake (g/1000 kcal)	55.2	8.5	54.3	8.3	56.2	8.5	**0.04**
Vitamin D intake (μg/1000 kcal)	9.4	5.9	8.8	5.6	10.0	6.1	0.09
Calcium intake (mg/1000 kcal)	321	119	297	113	346	119	**<0.01**
Smoking status (*n*, %)							**<0.01**
Non-smoker	168	54	27	17	141	90	
Ex-smoker	107	34	98	62	9	6	
Current smoker	39	12	32	20	7	4	
Drinking habit (*n*, %)							**<0.01**
Non-drinker	180	57	49	31	131	83	
Less than 20 mg/day	70	22	47	30	23	15	
20–40 mg/day	61	19	58	37	3	2	
More than 40 mg/day	3	1	3	2	0	0	

Note: *p*-values were from the Student’s *t*-test for continuous variables and the chi-square test for categorical variables. *p*-values < 0.05 are highlighted in bold. Abbreviations: BMI, body mass index; GS, grip strength; CC, calf circumference; BW, body weight; eGFR, estimated glomerular filtration rates; 25(OH)D, 25-hydroxyvitamin D; PTH, parathyroid hormone; SD, standard deviation.

**Table 2:** Comparison of characteristics between low- and high OSI groups according to sex.

	Male					Female				
	Low-OSI (*n* = 79)		High-OSI (*n* = 78)			Low-OSI (*n* = 76)		High-OSI (*n* = 81)		
	Mean (*n*)	SD (%)	Mean (*n*)	SD (%)	*p*-Value	Mean (*n*)	SD (%)	Mean (*n*)	SD (%)	*p*-Value
Age (y)	73.2	6.3	70.1	4.7	**<0.01**	73.2	6.9	71.1	5.3	**0.04**
Height (cm)	161.0	17.4	164.1	6.0	0.14	148.2	5.6	147.6	17.0	0.77
Weight (kg)	60.4	8.8	66.5	8.7	**<0.01**	49.9	6.1	53.5	6.8	**<0.01**
BMI (kg/m^2^)	22.7	2.9	24.7	2.9	**<0.01**	22.7	2.6	23.6	2.5	**0.04**
Systolic blood pressure (mmHg)	144	18	142	18	0.50	143	21	142	17	0.64
Diastolic blood pressure (mmHg)	78	12	79	11	0.58	77	13	76	10	0.64
Fasting plasma glucose (mg/dL)	101.9	15.6	104.0	27.5	0.56	93.3	10.5	97.7	18.1	0.06
Serum calcium concentrations (mg/dL)	9.28	0.33	9.29	0.30	0.78	9.31	0.34	9.36	0.33	0.36
Serum 25(OH)D concentrations (ng/mL)	27.2	7.2	28.7	8.4	0.23	24.4	8.3	22.9	5.9	0.20
Intact PTH (pg/mL)	46.4	18.5	43.8	15.2	0.34	51.7	20.5	49.4	17.4	0.44
CC/BW (cm/kg)	0.55	0.05	0.53	0.05	**<0.01**	0.64	0.05	0.61	0.05	**<0.01**
GS/BW (kgf/kg)	0.58	0.10	0.58	0.10	0.87	0.43	0.08	0.45	0.08	0.09
eGFR (mL/min/1.73 m^2^)	70.5	16.3	72.2	14.0	0.48	71.6	15.7	71.9	13.5	0.89
Osteo-sono assessment index	2.49	0.16	3.01	0.27	**<0.01**	2.15	0.10	2.51	0.18	**<0.01**
Total energy intake (kcal/day)	2016	590	2151	660	0.18	1745	598	1687	538	0.52
Protein intake (g/1000 kcal)	14.8	2.9	15.1	3.6	0.58	16.2	3.6	16.4	3.7	0.78
Fat intake (g/1000 kcal)	22.4	6.0	22.5	5.6	0.85	25.5	6.5	25.6	5.3	0.88
Carbohydrate intake (g/1000 kcal)	55.5	8.2	53	8.4	0.06	56.6	9.4	55.9	7.7	0.62
Vitamin D intake (μg/1000 kcal)	8.5	4.6	9.2	6.5	0.39	9.8	5.8	10.1	6.4	0.75
Calcium intake (mg/1000 kcal)	285	104	309	122	0.18	354	121	340	118	0.47
Smoking status (*n*, %)					0.83					0.52
Non-smoker	15	19	12	15		69	91	72	89	
Ex-smoker	48	61	50	64		5	7	4	5	
Current smoker	16	20	16	21		2	3	5	6	
Drinking habit (*n*, %)					0.27					0.63
Non-drinker	29	37	20	26		62	82	69	85	
Less than 20 mg/day	25	32	22	28		13	17	10	12	
20–40 mg/day	24	30	34	44		1	1	2	2	
More than 40 mg/day	1	1	2	3		0	0	0	0	

Note: *p*-values were from the Student’s *t*-test for continuous variables and the chi-square test for categorical variables. *p*-values < 0.05 are highlighted in bold. Abbreviations: BMI, body mass index; GS, grip strength; CC, calf circumference; BW, body weight; eGFR, estimated glomerular filtration rates; 25(OH)D, 25-hydroxyvitamin D; PTH, parathyroid hormone; SD, standard deviation.

**Table 3:** Interaction between sex and calcium intake levels on the OSI.

		Low Calcium Intake		High Calcium Intake				
Dependent Variable	Sex	Mean	SE	Mean	SE	*p* for Sex ^a^	*p* for Calcium Intake Levels ^b^	*p* for Interactions ^c^
OSI	Male	2.61	0.04	2.72	0.03	**<0.01**	0.28	**<0.01**
	Female	2.44	0.04	2.39	0.03			

Notes: Analyses were adjusted for age, body mass index, grip strength divided by body weight, estimated glomerular filtration rates, drinking habits, the smoking status, intact parathyroid hormone, and the serum 25-hydroxyvitamin D concentrations. *p*-values < 0.05 are highlighted in bold. ^a^: *p*-value of the main effect of sex; ^b^: *p*-value of the main effect of calcium intake levels; *c*: *p*-value of the interaction test between sex and calcium intake on the OSI; Abbreviations: OSI, the osteo-sono assessment index; SE, standard error.

**Table 4:** Multiple regression analysis of the serum calcium concentrations as a dependent variable according to sex.

	Total			Male			Female		
Variables	Non-Standardized β	Standardized β	*p*-Value	Non-Standardized β	Standardized β	*p*-Value	Non-Standardized β	Standardized β	*p*-Value
Age	−0.01	−0.12	**0.03**	−0.01	−0.10	0.23	−0.01	−0.13	0.10
BMI	0.00	−0.01	0.80	0.00	0.02	0.78	−0.01	−0.06	0.45
Drinking habits	−0.05	−0.12	0.05	−0.04	−0.11	0.19	−0.06	−0.08	0.32
Smoking status	−0.01	−0.01	0.86	−0.02	−0.03	0.71	0.05	0.08	0.36
Intact PTH	0.00	−0.20	**<0.01**	0.00	−0.23	**<0.01**	0.00	−0.17	**0.03**

Notes: β means a partial regression coefficient of each independent variable to the serum calcium concentrations. *p*-values < 0.05 are highlighted in bold. Abbreviations: BMI, body mass index; PTH, parathyroid hormone.

**Table 5:** Multiple regression analysis of the OSI as a dependent variable according to sex.

	Total			Male			Female		
Variables	Non-Standardized β	Standardized β	*p*-Value	Non-Standardized β	Standardized β	*p*-Value	Non-Standardized β	Standardized β	*p*-Value
Age	−0.01	−0.14	**0.01**	−0.02	−0.26	**<0.01**	−0.01	−0.31	**<0.01**
BMI	0.04	0.35	**<0.01**	0.04	0.32	**<0.01**	0.02	0.28	**<0.01**
eGFR	0.00	−0.10	0.06	0.00	−0.03	0.73	0.00	−0.23	**0.02**
Drinking habits	0.07	0.16	**<0.01**	0.03	0.08	0.35	−0.01	−0.03	0.73
Smoking status	0.11	0.21	**<0.01**	0.04	0.07	0.33	0.09	0.18	**0.03**
GS/BW	0.76	0.25	**<0.01**	0.05	0.02	0.85	0.55	0.19	**0.04**
Intact PTH	0.00	−0.12	**0.01**	0.00	−0.09	0.22	0.00	−0.11	0.16
Serum 25(OH)D	0.00	0.09	0.06	0.01	0.16	**0.04**	0.00	−0.03	0.74
Calcium intake	0.00	0.07	0.14	0.00	0.24	**<0.01**	0.00	−0.08	0.27

Notes: β means a partial regression coefficient of each independent variable to the OSI. *p*-values < 0.05 are highlighted in bold. Abbreviations: BMI, body mass index; eGFR, estimated glomerular filtration rates; GS/BW, grip strength divided by body weight; PTH, parathyroid hormone; 25(OH)D, 25-hydroxyvitamin D.

## Data Availability

Data in the present study are available upon reasonable request from the corresponding author. Data are not publicly available due to privacy and ethical policies.
